# Expanding worldviews on psychometric analysis of measurement tools in health professions education and research

**DOI:** 10.1002/ase.70053

**Published:** 2025-06-11

**Authors:** Michelle D. Lazarus, Mahbub Sarkar, Claire Palermo, Sze‐Ee Soh, Melanie K. Farlie

**Affiliations:** ^1^ Centre of Human Anatomy Education (CHAE), Department of Anatomy and Developmental Biology, Biomedical Discovery Institute, Faculty of Medicine, Nursing, and Health Sciences Monash University Clayton Victoria Australia; ^2^ Monash Centre for Scholarship in Health Education (MCSHE), Faculty of Medicine, Nursing, and Health Sciences Monash University Clayton Victoria Australia; ^3^ Faculty of Medicine, Nursing, and Health Sciences Monash University Clayton Victoria Australia; ^4^ Department of Physiotherapy, Faculty of Medicine, Nursing, and Health Sciences Monash University Frankston Victoria Australia; ^5^ Rehabilitation, Ageing and Independently Living (RAIL) Research Centre, School of Primary and Allied Health Care Frankston Victoria Australia

**Keywords:** anatomy and medical education, assessment, psychometrics, worldviews

## Abstract

Worldviews influence research—from design to interpretation and reporting. Historically, psychometrics has been predominantly situated within a positivist paradigm, while social research has often aligned with interpretivist or critical paradigms. However, emerging perspectives in the philosophy‐of‐science are challenging this rigid alignment, inviting a more nuanced understanding of the relationship between worldviews and methodologies in the health professions. This article explores both historical and emerging worldviews related to psychometrics—asking readers to reconsider assumptions and preconceived ideas about which worldview applies to this field of research. Furthermore, this article challenges the psychometrics community to consider how the field can occupy seemingly competing worldviews and approaches, including those that have historically been more commonly associated with qualitative research—such as critical theory (under the broader umbrella of critical inquiry) and reflexivity. Using illustrative case examples applicable to the anatomical sciences and health professions, we discuss the evolution of perspectives on psychometrics and its relationship to worldviews through the lenses of classical test theory and item response theory to the contemporary paradigm of “critical psychometrics”.

## INTRODUCTION

Whether consciously or not, health professions educators and researchers bring certain beliefs and philosophical assumptions into their practice. These beliefs are not arbitrary but deeply influenced by personal experiences, professional backgrounds, and prior education. Such beliefs, often called worldviews, philosophies, paradigms, and grand theories, play a significant role in shaping choices regarding which problems to investigate, the questions they pose, and the methods they employ for data collection and analysis.^1^, ^2^

Tensions can arise between a researcher's personal worldview and the methods and methodologies they engage to evaluate health professions education (HPE) and conduct HPE research. For instance, a desire to measure learners' competencies—typically associated with a scientific and objective method and positivist/post‐positivist worldview—may conflict with a researcher's perceptions that such competencies are complex and contextually determined. The latter perspective suggests the researcher maintains an interpretivist worldview that is historically tied to more qualitative methods.

The focus of contemporary HPE on empirical observations and a biomedical model can contrast against the complex psychosocial and educational contexts being studied. These tensions are critically important for the HPE field to consider given the impact of the field on both HPE research and practice. This discursive article provides an overview of key terms related to worldviews and research methodologies, describes tensions that can present between these two concepts, and suggests a way forward for those engaging with and reviewing HPE research.

### Worldviews in research

Worldviews influence how education scholars and researchers conceptualize measurement and—more broadly—how individuals perceive their own and others' measurement practices. In this way, worldviews influence individuals' interpretations of practice in the field, the research questions they ask, and the approaches and methodologies they select. Tensions can arise when an individual's worldview is incongruent with the approaches or methodologies used in practice. These tensions can lead to misinterpretation or dismissal of others' work or a dissonance between how the researcher conceptualizes the topic being studied and the research method used in the study. Having a deeper understanding of internal coherence (i.e., the relationship between worldviews and the educational scholarship and research process^3^) supports practitioners in their work and their considerations of others' contributions to the field. This ultimately impacts how learners are assessed and can be used to make high‐stakes decisions such as decisions on progression through their health professions degree.

In this article, we explore the tensions between worldviews and the approaches or methodologies used in the scholarship of education and research by focusing on analyzing and interpreting the psychometric properties of measurement tools employed in HPE settings. While there are multiple worldviews and associated methods for exploring a construct of interest, psychometric studies have dominated key HPE journals over the past decade.^2^ This discursive article brings to light the explicit relationships between worldviews and the establishment of measurement tools' psychometric properties by reviewing the different worldviews and their historically associated research methods. In doing so, we make an argument for having all researchers, regardless of one's worldview, engage in reflexive practice throughout the research process. In this article, we lay bare the tensions that can arise between personal worldviews and research methods to support a more effective interpretation of the literature and its subsequent application into HPE practice.

#### Defining worldviews and the relationship with truth: A brief overview

Worldviews, as comprehensively described in the text by Varpio et al.^4^, encompass perspectives on the nature of reality (ontology), the process of knowing (epistemology), how knowledge is accessed or constructed (methodology), and the values inherent in research (axiology). Ontology pertains to individuals' assumptions about the fundamental nature of reality, such as whether there exists a single objective reality or multiple subjective realities. Epistemology represents our understanding of what can be known about reality and how knowledge is acquired. Ontological and epistemological assumptions shape researchers' choice in the methodologies employed to access or create knowledge. Additionally, they are informed by sets of values (axiology) that influence why certain types of research are valued and considered worthwhile by an individual.

Prominent worldviews guiding health professions and science education research include positivism/post‐positivism, interpretivism, critical inquiry, and pragmatism. Contemporary HPE tends to focus on empirical observations and a biomedical model, which aligns most strongly with positivism/post‐positivism. However, depending on the research question a study attempts to address, different worldviews have been historically viewed as more appropriate than others.^5^ Table 1 provides an overview of commonly described worldviews and related methodological approaches. Worldviews influence, and are influenced by, how truth is conceptualized by the researcher and guide the selection of methodological approaches to explore research questions and aims.

**TABLE 1:** Summary of research worldviews, key assumptions, and related methodological approaches.

Worldview	Key assumptions	Common methodological approaches	Example research question
Positivist/Post‐positivist	Measurable objective reality Knowledge empirically derived Scientific method is paramount	Quantitative Study Design Meta‐analytic reviews Experimental studies Non‐experimental studies (e.g., retrospective data analysis) Psychometrics	What are the anxiety levels of first‐year medical students due to their first human donor dissection experience?^6^
Interpretivism	Socially constructed reality Understanding is based on subjective experiences Privileges participant voices Researcher reflexivity Nuanced descriptions & interpretation problems Context & meaning are central	Qualitative Study Design Case studies Ethnography Narrative inquiry Grounded theory Qualitative description Phenomenology Qualitative Data Collection Methods: Interviews Document review Observations	How do medical students experience emotions during the first human donor dissection laboratory?^7^
Pragmatism	Focus on practical consequences & what works Truth is what works at the time Combines both objective and subjective perspectives	Mixed‐methods integrating quantitative and qualitative Convergent Explanatory Sequential Exploratory sequential	How do differing healthcare professional anatomical curricula and their frameworks influence perceptions of medical ethics for students in medical, nursing and allied health programs?^8^
Criticalism	Reality is shaped by social, political, cultural, economic, ethnic, and gender values Aims to critique and change society	Co‐production, participatory action research, visual methods Photovoice^9^, ^10^ Video‐reflexive ethnography^11^ Critical discourse analysis Critical review Critical ethnography Decolonising methodologies	How do dominant anatomy education approaches and resources reinforce students' perceptions of disabled people?^12^, ^13^, ^14^

##### Positivism and post‐positivism

Positivism holds that reality is constant, singular, tangible, measurable, and constructed through cause‐and‐effect relationships.^15^ Researchers engaging with the positivist worldview seek to explain and predict universal features of the research problem, privileging the hypothetico‐deductive model.^15^, ^16^

Post‐positivism extends from positivism by acknowledging the limitations in fully understanding such truths due to “imperfect” measurements.^16^ Post‐positivism recognizes that reality is inherently incomplete because it is derived from observations shaped by human bias.^17^

These worldviews typically fall within scientific approaches and are particularly valuable when explaining the effectiveness of interventions or educational tools^2^ (Table 1).

Many researchers working within these paradigms may not explicitly state their philosophical assumptions as positivism or post‐positivism, though they typically start with a theory, develop hypotheses, and use empirical observation to predict patterns, including causality ‐ leading to an implicit recognition that these worldviews were employed. Importantly, human experience and perceptions is viewed through a “deficit” lens (e.g., bias) in positivist/post‐positivist worldviews because the focus is on identifying objective (or close to it) truths.

**FIGURE 1:**
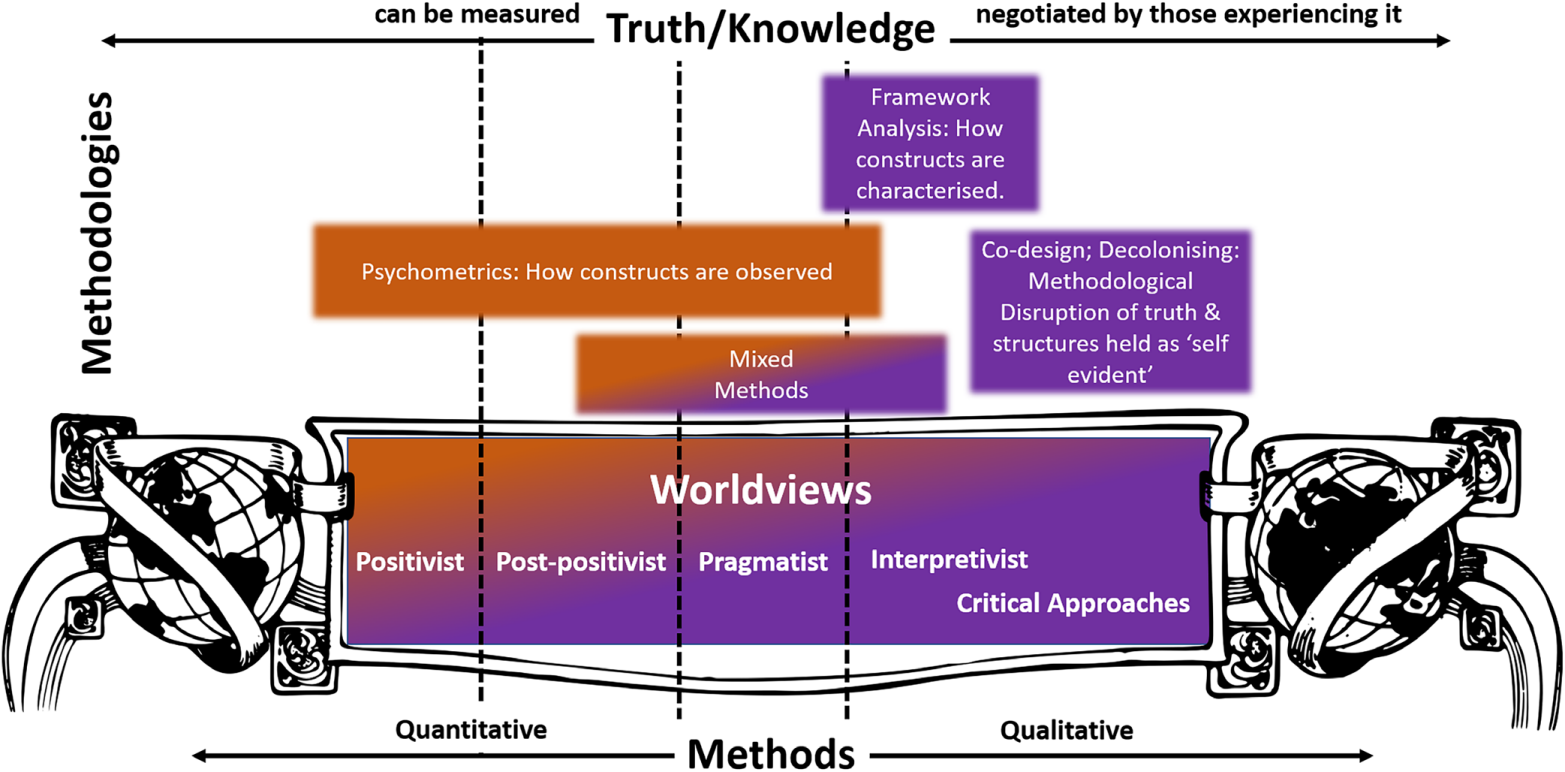
The historical relationship between worldviews, methods, and methodologies. Historically, methods were more rigidly linked to different worldviews. Psychometrics were linked to more positivist/post‐positivist worldviews (orange box), emphasizing that truth and knowledge are measurable. Framework analysis and Co‐Design were linked to more qualitative approaches (purple boxes), which relates to interpretivist and critical approaches worldviews. These worldviews are more strongly tied to a view that truth and knowledge are negotiated by those experiencing it. Pragmatist worldviews are historically tied to mixed‐methods (orange to purple gradient box). Contemporarly, the relationship between these methods/methodologies remains unclear, and, thus, the boxes representing them have fuzzy borders.

Common methodological approaches utilized within positivism/post‐positivism include meta‐analytic reviews (focusing on the quantitative synthesis of results), experimental (e.g., randomized control trials) and non‐experimental studies (where variables are measured, but controls are not implemented such as for retrospective cohort studies), and—importantly—psychometric studies^18^ (Figure 1, orange box). Experimental methodologies, such as randomized controlled trials, may investigate the effectiveness of interventions in controlled or ideal environments (often associated with positivism) or explore their real‐life applicability and effectiveness for specific populations (often associated with post‐positivism). Non‐experimental methodologies are primarily observational studies where researchers explore and describe patterns and differences found in the study. Measurement studies investigating psychometrics are considered non‐experimental research.

Positivism is the historical proverbial “gold standard” for HPE research.^4^ Contemporarily, however, the field of HPE research is expanding to accept other worldviews and definitions of truth.^19^, ^20^

##### Interpretivism

In contrast to positivist and post‐positivist worldviews, interpretivism recognizes a multiplicity of reality and contends that understanding the social world requires full immersion within its social context.^21^ Instead of striving for objectivity, those engaging in interpretivist worldviews prioritize the cultivation of subjective meanings, including participants' beliefs and intentions. Thus, researchers in these approaches perceive knowledge as subjective and evolving through social interactions.^22^ The significance of this worldview lies in acknowledging the influence of individual experiences and interpretations on knowledge formation. It acknowledges the symbiotic relationship between research (including researchers and participants) and practice, where each informs and shapes the other. Researchers adhering to interpretivism are guided by their interests and beliefs, impacting their research and interpretation approaches.

Unlike positivism and post‐positivism, interpretivist researchers typically state their positionality, with this research approach being increasingly engaged to explore the nuanced role that different teaching practices have on learning (Table 1).

##### Pragmatism

Pragmatists acknowledge both singular and multiple realities, viewing knowledge as constructed based on their interactions with dynamic reality.^23^ As a grand theory, pragmatism assumes that truth is contextual and influenced by social, political, economic, and historical factors. It emphasizes that what is considered true can differ based on specific circumstances, periods, and the individuals involved, shaped by their perceptions and experiences within those contexts. Researchers who adopt a pragmatist approach see knowledge as temporary and changeable. Therefore, pragmatist inquiry is viewed as an ongoing process of investigation and action, leading to constant evaluation of their understanding of the world and adjustment of their behaviors. Truth is seen as provisional and contextual, encouraging dialogue between different approaches and one that embraces pluralism.^24^

Pragmatists often adopt a mixed‐methods approach, rigorously integrating qualitative and quantitative methods to address research questions,^25^ and an analysis that integrates the two strands into coherent conclusions or inferences.^26^ By doing so, the study can offer a more comprehensive and meaningful understanding of the research topic than either qualitative or quantitative methods alone are likely to provide. Many mixed‐methods typologies are available, but the prominent ones include convergent, explanatory sequential, and exploratory sequential.^26^

##### Critical approaches

Critical approaches seek to critique and change factors that oppress individuals, questioning prevailing assumptions and highlighting power dynamics and privilege.^27^, ^28^ Those engaging critical approaches emphasize perspectives of marginalized groups for empowerment and social justice, challenging existing ways of knowing and the conditions and structures across society that give rise to them. While critical approaches may align with a subjectivist epistemology, suggesting that knowledge is socially constructed, they also posit that competing groups continually challenge what is considered knowledge and how it is constructed.^1^, ^29^ In this way, interpretations of experiences are perceived as unequal and vary among groups due to the influence of power dynamics, social positioning, and underlying ideologies shaping collective perspectives.

Since interpretations of experiences vary among groups due to power dynamics, social positioning, and underlying ideologies, critical approach methodologies are iterative. This involves relational and reflexive dialogues between researchers and participants to foster change^1^ and inherently necessitates transdisciplinary approaches to interpret social experiences from multiple perspectives.^29^ Examples of critical methodologies include co‐production, participatory action research, visual methods (e.g., photovoice, video‐reflexive ethnography), critical discourse analysis, critical review, critical ethnography, and decolonizing methodologies.^28^ Indeed, there is a call for more consideration of critical approaches in HPE^12^ and the field of psychometrics.^29^, ^30^

#### Worldviews in psychometric studies

In the broadest sense, psychometrics is the study of the properties of tools designed to measure knowledge, attributes, and skills.^31^ While historically, the field of psychometrics has its origins in psychology, establishing the psychometric properties of measurement tools is now an integral part of healthcare education practice and research.^32^ Establishing the psychometric properties of a measurement includes the examination of validity (e.g., does the measurement tool measure the construct of interest?), reliability (e.g., how repeatable are the measurements obtained with a measurement tool?), and responsiveness (e.g., does the measurement tool detect changes over time?).^31^ For these reasons, psychometrics is predominantly and historically tied to positivist worldviews.

In practice, however, the researcher's alignment between worldview and methodologies is not as bounded or well‐defined in its ties to positivism. Psychometrics can have fuzzy borders where overlaps between methods and worldviews can occur (Figure 1).^30^ While psychometrics focuses on the development and testing of scales designed for measuring a variety of observable human cognitions, emotions, and behaviors—the historical lens applied to psychometrics was exclusively based on the principles of being scientific and, thus quantitative, value‐free, and objective (positivist worldview). Contemporary psychometric approaches expand this view by re‐evaluating the extent to which these principles apply when humans are involved (leaning toward a more post‐positivist worldview), and some are beginning to challenge the field to expand the consideration of social influences on measurement to include power dynamics (e.g., critical theory).^30^ These more contextual views of measurements increasingly lend themselves to integrating psychometrics methods with pragmatist, interpretivist, and critical inquiry worldviews.

#### Applying psychometrics in health professions education

Health professions education is increasingly recognized as situated within a complex system, with learning influenced by various individual factors, including prior learner experiences. Relational aspects (e.g., interactions with peers and educators) also have an impact. Furthermore, HPE and HPE research are recognized as being influenced by society, systems, and structures within which education occurs. In this way, those entering the learning environment will likely think, act, and feel differently as they progress through the learning journey toward their degree; in other words, a learner is not the same person leaving a classroom as when they entered it. This acknowledgment of complexity and changeability has resulted in recent criticisms of HPE psychometric studies situated in a positivist paradigm due to their inadequate treatment of context, the role of the researcher, and research participants in the research process, and the complexities of the education systems investigated.^18^

Strategies identified to address these positivist limitations in educational assessment (e.g., learner “testing”) stem from the more contemporary post‐positivist psychometrics era. These strategies consider assessment in two ways: (1) as a judgment or (2) as a system.^33^ It is increasingly acknowledged that human judgment is inextricably linked to assessment even in its most basic structural forms,^33^ and the influences of the system are similarly inseparable from the construct being measured or evaluated. In this way, the historical positivist approach to psychometrics is increasingly questioned—and arguably rightly so.

### Measurement theories used in psychometrics

Worldviews influence the methods or approaches engaged in psychometrics. Classical test theory (CTT) has been the foundation of measurement theory and the scale development process for over 80 years. CTT considers a construct through a more positivist lens. In doing so, CTT postulates that the construct of interest is stable in a given population and that each person engaging with the measurement tool is “equal”—aligning with the objectivity typical of this worldview. An alternative psychometric approach is found with Item Response Theory (IRT), which considers a construct through a more post‐positivist worldview. IRT postulates that a response to an item on a measurement tool is a function of the item's difficulty and the person's ability—thus IRT considers contextual influences in the measurement.

CTT and IRT are both approaches to measure constructs, such as abilities or attitudes, by analyzing individual test items to evaluate their quality (accuracy) and contribution to the overall assessment (weighting). Furthermore, both approaches use methods to evaluate the test measurements' reliability (reproducibility) and validity (accuracy).

As contemporary psychometrics scholars explore the field's evolution, an area identified as an emerging worldview is critical inquiry approaches.^29^, ^30^ With a key tenant of psychometrics being “fairness”,^34^ critical inquiry provides a lens by which researchers can consider fairness outside of the typically applied differential item function/DIF (e.g., gender, ethnicity, age, etc.) toward one that considers the intersectionality of these factors and how these “hidden” elements impact on the measurements undertaken. An example of this includes the Transdisciplinary Philosophy‐of‐Science paradigm which engages with psychometrics through a critical realist lens.^29^ When applying a criticalism worldview lens to scales and evaluations, researchers can ask whether the standards by which the measurement is designed (and the individuals responsible for identifying and setting these standards) are representative of the population being evaluated and whether the interpretations of the scales are fair and appropriate in this context.^35^ By applying elements of critical inquiry to psychometrics, we can begin to explore the extent to which scales can and should be used to measure psychological constructs in different populations—and whether elements of a scale may need to be adjusted to appropriately measure the construct of interest in the study context. There are a variety of risks when we do not consider key tenants of critical inquiry (e.g., power dynamics, social positioning, and underlying ideologies). These risks can include measurement challenges such as incorrect assumptions and interpretations of the measurement tools. There are also broader societal risks when critical theories are excluded from evaluation approaches including the acceleration and further entrenching of bias into our teaching, further marginalizing our learners—and even communities ‐ as assessment tools are used more broadly on a global scale.

Consider the examples of intelligence quotient (IQ) testing. While IQ testing appears to have validity and reliability across some populations, a lack of considering criticalism has resulted in the misuse and misinterpretation of the results—resulting in harm.^36^ The idea that intelligence is a separable entity, one that is divided into thinking and emotion (e.g., emotional intelligence vs. IQ)—is a concept that is itself problematic in many cultures including some First Nations Australian cultures.^37^ Yet, such tests are used to define benefits and supports, disabilities, and even jobs across the globe. A critical lens asks us to consider the extent that IQ testing is valid and reliable in different cultures, and asks psychometricians to also consider for whom this type of testing would be less reliable and valid. In this way, a critical lens can support the mission of the research to ensure that the instrument is measuring what is intended, and can guide us on the appropriateness of using a particular instrument in a given context—and thus represents a necessary evolution in the field of psychometrics. This is similar to the field's journey in considering “validity” from a once‐and‐done approach (aligning more with a positivist worldview) to one that considers an evidentiary chain,^33^ aligning more with a pragmatist worldview.

Palawa Professor Maggie Walter highlights in her book Indigenous Statistics^38^—that there is no truly objective quantitative research. There are implicit and explicit decisions by humans, who carry with these perspectives a bias (including racism). Professor Walter highlights how these decisions lead us to interpret psychometric study statistics as facts, particularly when peer‐reviewed and published, which amplifies some perspectives (e.g., the researchers) over others (e.g., the population being studied). Further entrenching this in our society is the tendency to translate these numerical “facts” into policy. Consider this study^39^ that explores the emotional competencies of students in culturally and linguistically diverse (CALD) backgrounds, compared to those who are non‐CALD. This framing privileges one cultural group as the reference standard for all “other” cultural groups. From a critical perspective, such assumptions present fundamental issues in the validity of the measurement tool. This particular study concluded that non‐CALD students performed significantly higher on the emotional competency scale (developed in that population) than those who were from CALD backgrounds. Critical inquiry asks us to consider the philosophical and social consequences for interpreting the results of this study in this way, where a scale developed with one socio‐cultural group is assumed to measure the same construct(s) in other socio‐cultural groups. Criticalism also asks us to consider which group is appropriate to serve as the reference population in a study.

While in its early stages, a psychometric‐adjacent field is emerging which includes some key tenants of critical theory—that of QuantCrit.^40^ The use of the QuantCrit approach for statistical model development has been characterized as:

explor[ing] the additional information that including interaction terms for demographic variables provides. By having an interaction term for gender and race, a model can predict the impacts of sexism and racism in ways that are not merely additive.

Thus, while psychometrics has historically been tied most strongly to objective facts and figures (e.g., positivist worldview), the field is beginning to consider critical methods that account for the context within which these figures apply—and the context of those determining the psychometric methodology and modeling explored.^29^ Indeed, such an approach, which could be termed “critical psychometrics,” may help psychometricians expose power dynamics that might be at play *before* a scale is used (arguably misused) and help the field consider power and privilege in the interpretation of the results of psychometric studies.

In this way, worldviews influence psychometrics. There are practical examples of this such as the USA‐based Scholastic Aptitude Test (SAT) as an example of a scale developed using CTT methods and the Assessment of Physiotherapy Practice (APP) developed using IRT.^41^ Each example helps illustrate how psychometric approaches differ based on their relationship with the measured person or population.

#### Assumptions in classical test theory (CTT)

Fundamental assumptions for CTT align most strongly with positivism, whereby a test score is comprised of a true score and an error score, with the error score occurring randomly. CTT assumes that across all test items, difficulty and distribution, the true score will converge toward a normalized distribution.^42^, ^43^ Thus, the focus of analysis in CTT is on *total* test scores and *overall* test reliability (not its validity),^44^ because the true score refers to the average score that would be obtained if we administer the test/instrument multiple times.^42^ This approach is recognized when the test/instrument is designed to measure the total score of a construct using a set of complementary items. In this approach, longer instruments tend to be more reliable because more items mean a greater likelihood of measuring the underlying construct. For example, a scale designed to measure an individual's IQ has items that measure comprehension, reasoning, memory, and processing. However, the estimate of the person's intelligence is based on the total score on the test, not performance on individual items. Similarly, the Scholastic Assessment Test (SAT), a standardized test that measures U.S. students' readiness for college, also uses CTT in its design. The SAT includes numerous items that assess math, reading, and writing, acknowledging that aptitude is multidimensional and not unidimensional. A person's SAT score represents an overall performance—with sub‐scores provided for each domain (or dimension). Such a multidimensional CTT‐designed test can give an overall composite scholastic aptitude score across all dimensions and within each dimension—but an individual cannot know their aptitude by looking at a single item from any section of the SAT. Despite each item evaluating an aspect of knowledge, individual items alone ‐ in isolation, a single item cannot provide insight into the whole.

While many forms of reliability evidence (i.e., the extent a scale produces consistent results and is free from random error) exist for CTT‐designed scales and tests, internal consistency reliability (i.e., the degree of inter‐relatedness among items in a scale^45^) is commonly reported using Cronbach's alpha. It is expressed as a reliability coefficient and can be interpreted according to six levels from “unacceptable” to “consider shortening the scale”.^46^ ^(p109)^ For example, suppose a test has a reliability coefficient of 0.65. In that case, it means that 65% of the variance in the test scores across a population is due to true differences in the trait/construct being measured, and 35% is due to measurement error. De Vellis^46^ states that a scale with a reliability coefficient of 0.65 has minimally acceptable internal consistency reliability. Given that CTT focuses on the total scores produced by the instrument (not the instrument itself),^42^ strong evidence is needed for the reliability and validity of instruments to be transferred across occasions and populations before we can have confidence in its usefulness as a measure for a new population. For example, while the SAT may be a reliable and valid measure of aptitude in U.S. students, it may not perform in the same way for students from a different background.^47^

Another core tenant of CTT‐developed scales is the assumption that reliability is influenced by random error instead of contextual differences (e.g., age, gender). Let's consider a construct that has been explored within the HPE field—that of uncertainty tolerance. Uncertainty tolerance is defined as how individuals (in this case learners) respond to uncertainty across how they think, feel, and act.^48^ Multiple governing bodies list effectively managing uncertainty as a health profession graduate attribute,^49^, ^50^, ^51^, ^52^ thus measuring how well HPE curricula are impacting learners uncertainty tolerance could be of great interest. Box 1 illustrates how CTT has influenced the development and testing of this construct in HPE. The limitation of CTT not accounting for contextual differences has rendered many existing uncertainty tolerance scales unfit for the purpose of evaluating HPE learners (Box 1), despite being adequate for measuring the same construct in experienced doctors.^53^, ^54^

BOX 1 **Evaluating uncertainty & ambiguity tolerance in health professions education (HPE).**

Measurement justification: Increasingly represented as a health care graduate attribute.

**Brief psychometric history**

Frenkel‐Brunswick scale^55^ is credited with developing the first scale evaluating the construct of “intolerance of ambiguity” which in contemporary research is often synonymous with uncertainty tolerance.^56^ The Frenkel‐Brunswick scale, and the later iteration—the Budner scale^57^—are the scales most often adapted and evaluated for psychometric properties within HPE.

Recent reviews have highlighted that these scales, presumably developed using CTT, have inconsistent results across different populations^58^ and inconsistent validity evidence across studies and populations.^53^, ^56^, ^59^

Scale performance insufficiencies have been ascribed to the complexity of the construct and limited knowledge of the construct which is attributed to the lack of exploratory and qualitative studies and theory development. Recent work has focused on exploratory and qualitative studies to help elucidate these gaps, exploring the definitions of uncertainty in different populations, and what it means to be uncertainty tolerant.

**Historical approaches to uncertainty tolerance scale development in HPE: Classical Test Theory (CTT)**

- Over 20 different uncertainty tolerance scales have been tested and developed for use in HPE,^48^ all purported to measure the same construct.
- The validity evidentiary chain of uncertainty tolerance scales relies predominantly on exploring internal consistency (e.g., Cronbach's alpha).^54^
- Because of the underlying assumptions of  CTT, these uncertainty tolerance scales all need to be “revalidated” with each use, meaning validity evidence needs to be collected within each new context in which the scale is administered.
- In the uncertainty tolerance field of research, such “revalidation” resulted in evidence of differing validity across populations, namely between health professions learners and practising health professionals^53^ when using the uncertainty tolerance scales.
- Because the CTT approach typical of uncertainty tolerance scale development assumes that validity is influenced by random error and not contextual differences (e.g., differing perceptions of uncertainty across populations), these scales are not fit for purpose in high‐stakes assessments.^54^

**Potential future for uncertainty tolerance scale development in HPE: Item Response Theory (IRT) and Rasch analysis**

- A Rasch analysis was used to develop a scale measuring uncertainty tolerance about the healthcare environment, defined as “an individual's perceived inability to accurately predict the environment” (García‐Pérez & Yanes‐Estévez, 2022, p. p.761).^60^ In healthcare, environmental uncertainty can stem from funding variability, resource limitations and the complexity of the healthcare system regulations and governance.^61^
- For the research undertaken by García‐Pérez & Yanes‐Estévez,^60^ the context was the business environment. Scale items carried with them different ‘levels’ of uncertainty, with some items having greater uncertainty and others having less uncertainty (based on the research conducted leading to the scale development). A participant who *perceives* a low uncertainty for an item noted as having high uncertainty would indicate, a high uncertainty tolerance for that person on that item.
- Because this scale used IRT and Rasch analysis, the scale allows item level analysis for individuals resulting in the capacity to stratify individuals based on their level of uncertainty tolerance. Such a scale allows the researcher to ascertain the context relevant to this stratification (e.g., item person map) or sources of uncertainty that represent increasing levels of uncertainty. For instance, García‐Pérez et al.^60^ found that items related to “suppliers” represented less uncertainty than items related to the economy.

**Key take aways**

- Extensive qualitative and exploratory studies are necessary for effective engagement of psychometrics in scale development.
- IRT considers contextual factors in measurement, moving toward post‐positivist paradigms, and may be of more value in the complex context of Anatomy and HPE research.

Qualitative research conducted more recently, though following existing scale development, suggests that the variable validity between populations may be due to how individuals experience and perceive uncertainty.^62^, ^63^, ^64^ In summary—when it comes to uncertainty tolerance scales, context matters and a CTT approach to psychometrics may be ill‐fit for measuring this construct. Item Response Theory, however, may provide an alternative approach.

#### Assumptions in item response theory (IRT)

Fundamental assumptions for IRT are that the probability of a test/scale response is a function of the measured construct *and* the item's attributes.^65^, ^66^ In the case of Rasch (e.g., a single parameter model) there are further detailed assumptions such as: unidimensionality where only one latent trait is measured by the test items, local independence which indicates that responses to different items are independent of each other given a certain ability level, and monotonicity where the probability of a correct response increases as the ability level increases.^65^, ^66^ IRT falls under a broader category of “latent trait models” (LTM). Importantly, not all LTM psychometric approaches assume construct unidimensionality. For instance, multivariate Rasch analysis may account for multiple influences on the desired construct.^67^ As such, IRT analysis focuses on item characteristics and how each item interacts with the measured construct and with the individual being assessed or measured.^42^ In this way, an IRT‐developed test/scale considers how an individual responds to items on the scale, regardless of context.^42^, ^65^, ^66^ This means that individual items in a scale can have different levels of difficulty and discrimination. Mathematical models (e.g., Rasch models) estimate a person's individual ability related to the measured construct, based on the pattern of their item responses.^68^ For example, an item can be analyzed to determine its difficulty (e.g., for a person of average ability, how likely are they to answer correctly?), discrimination (e.g., how well does the item differentiate two individuals with different ability levels?) and the likelihood of a person simply guessing the right answer.^67^, ^69^, ^70^, ^71^

Due to the assumption that all items on the test can differ in difficulty and discriminate between individuals, assuming the items fit the (e.g., Rasch) model, a validated scale is considered invariant and provides comparable measures between groups without the need for revalidation (e.g., an instrument's validity is transferable across populations and contexts). An example of an IRT‐developed scale is the Assessment of Physiotherapy Practice (APP) scale developed using Rasch analysis to assess the entry‐level competence of physiotherapy students on clinical placements. All 20 items were invariant, indicating that the scale performed comparably across populations regardless of contextual factors related to the student, clinician, educator, or placement setting.^72^ An example of how IRT can, similarly, apply to the construct of uncertainty tolerance is included in Box 1. A Rasch‐developed uncertainty tolerance scale in HPE could measure an individual's capacity for managing uncertainty tolerance in different contexts (e.g., clinic versus classroom) and across those with differing levels of uncertainty tolerance. In such scales, we might find that some learners can manage uncertainty similarly to health professionals, and others may not. Currently, we can only make claims with existing population‐wide CTT scales (e.g., there is a significant difference between the population of healthcare professionals' performance on uncertainty scales and that of health profession learners).^56^

#### Value of IRT to HPE research & practice

**FIGURE 2:**
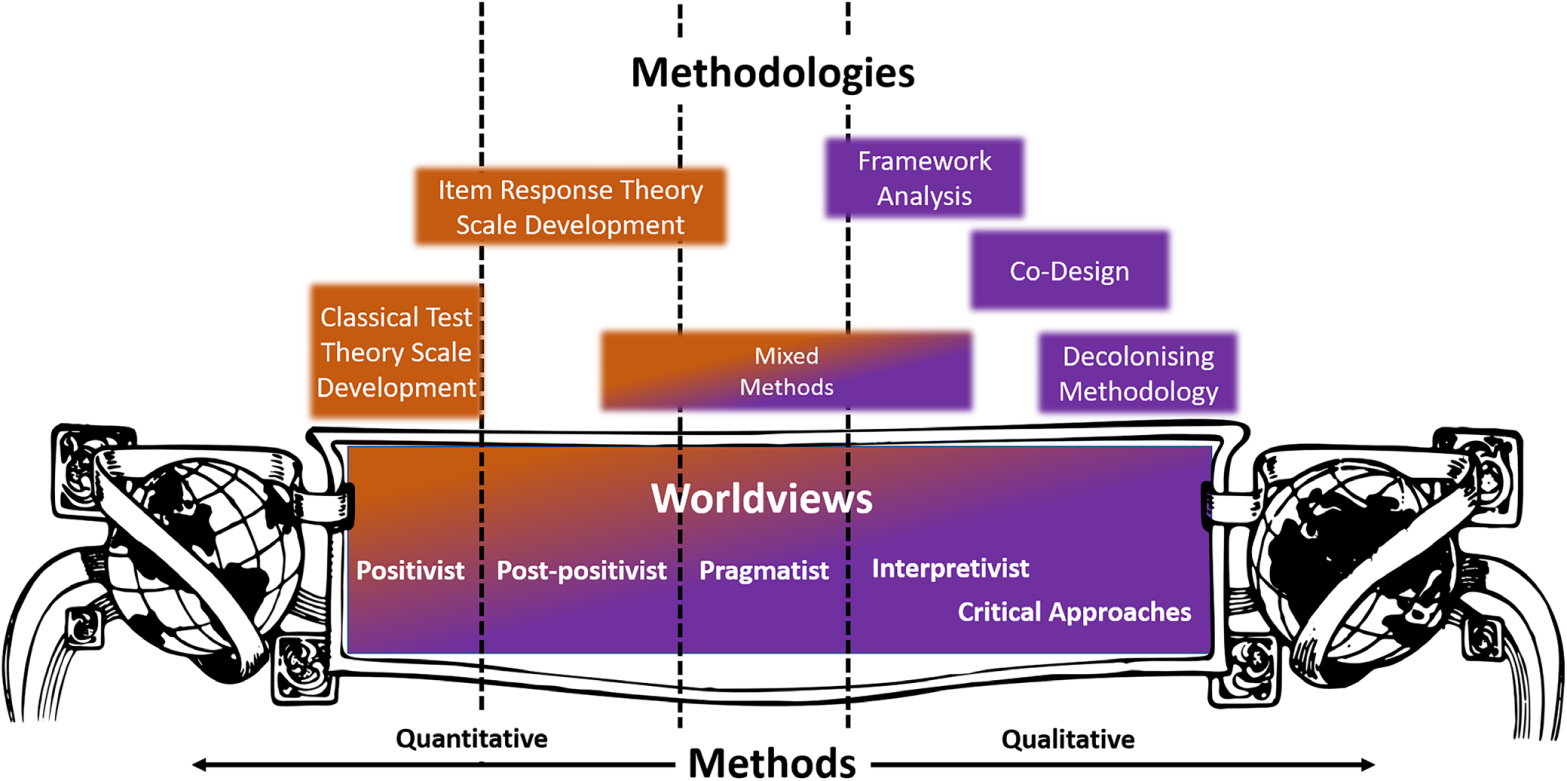
The historical relationship between worldviews and methods. Psychometrics has been historically represented by positivist worldviews, which rely on classical test theory (CTT). Contemporary psychometrics has progressed to include item response theory approaches to scale development, which can be linked to post‐positivist worldviews (orange box). Framework analysis and Co‐Design are linked to more qualitative approaches (purple boxes), which relate to interpretivist and critical approaches worldviews. Historical representations of worldviews and methods are along a linear continuum.

Rasch‐validated, IRT‐developed scales move toward the post‐positivist/interpretivist end of the worldview spectrum (Figure 2) because such scales consider context. In health professions education and research contexts, this could include an individual learner's experience and stage, both of which are shown to influence uncertainty tolerance.^73^

If the IRT approach described in Box 1 is applied to HPE, new uncertainty tolerance scales could be developed to measure learners' uncertainty tolerance at different career stages (from student to expert practitioner), geographical locations, clinical settings, and so on—all of which are shown to potentially impact an individuals' uncertainty tolerance.^58^ Using Rasch analysis, researchers could begin to unpack the relationship between the individual (and their demographic context) and their uncertainty tolerance in different scenarios instead of relying on the blunt instrument of population‐based conclusions typical of CTT. Such a new scale could be invaluable given the importance of developing uncertainty tolerance in HPE and the need to assess this to provide feedback about both the learner and the HPE program.

Researchers studying complex constructs within HPE are already engaging Rasch analysis for scale development and validation. These scales include the Feedback Quality Instrument (FQI), developed to consider contexts influencing feedback quality, such as the individual providing the feedback, the terminology used for the scale rating categories, and the measurement approach.^74^ This scale includes items on how well the feedback provider fostered learner agency and whether they created a psychologically safe feedback environment.

Another example is the aforementioned APP. While professional competence is a complex construct, Dalton and colleagues' work^41^ illustrated that a Rasch‐validated scale can measure a person's ability (i.e., practice competence) using items demonstrating one‐dimensionality (where the items in the scale measured one underlying construct). On the APP, their work demonstrated that items representing professional behavior and communication were the least difficult, while items related to complexity and uncertainty, such as patient planning and application of evidence‐based practice, were the most difficult. Therefore, students with the highest proficiency or competence in clinical practice will be able to satisfactorily achieve the most difficult items.

These examples illustrate how an IRT test/instrument/scale development approach can account for personal context and item difficulty in providing feedback to the assessed individual. Such IRT scale development approaches advance traditional approaches toward psychometrics. Arguably, IRT approaches represent a move from positivist, context‐independent, value‐free toward post‐positivist/interpretivist measurement assumptions. This is achieved by the introduction of models that consider construct complexity. Future work could further consider applying “critical psychometrics”—or lessons from critical inquiry—to psychometric approaches, such as those considered in the QuantCrit method^75^—further challenging us to consider who is defining the construct and who is involved with the scale development and interpretation, and who is left out or marginalized by this process. Future progress in this direction will need to explore the intersections between psychometric approaches, measurement theory, pedagogy, and worldviews. The writings of Usher are informative here, as they describe how grappling with this type of intersectionality requires a transdisciplinary philosophy‐of‐science paradigm.^29^

### The role of qualitative methods in scale development

No matter the scale development approaches a researcher chooses, scale development requires a rich understanding of the assessed construct. Typically, this means that extensive qualitative research ‐ and the related interpretivist worldviews ‐ may be necessary to capture the complexity of the construct.^76^, ^77^, ^78^ As we saw with the uncertainty tolerance construct, a lack of understanding of the construct in different contexts likely contributed to the differing validity evidence across populations. To address this gap, a series of qualitative research studies have helped capture the complex experiences of uncertainty in health professional learners in pre‐clinical^62^ and clinical settings.^54^, ^63^, ^64^ This qualitative approach can further inform uncertainty tolerance scale development, hopefully in a way that provides stronger evidence of reliability and validity.

Ultimately, when a holistic lens is applied to psychometrics, the notion that scale development and implementation are positioned exclusively within a “positivist” paradigm is dissolved. Scale development is informed by the interpretivist paradigm (Figure 2) and, depending on the theory engaged (and the type of scale or instrument used), the psychometric analysis may be considered anywhere along the worldview continuum from positivist to post‐positivist—and even interpretivist (in the case of critical psychometrics). Qualitative research is often used to deeply explore and understand a construct, which then informs scale development—and so on. In this way, a negative or judgmental critique of interpretivist or critical worldviews (and qualitative methods) could call into question the foundation of the scientific approaches often lauded in the field; by questioning the qualitative studies upon which the scales are built—we simultaneously question the scales themselves.

In practice, the positivist‐interpretivist research “cycle” is often synergistic, with each approach informing the other to develop a more comprehensive understanding of knowledge situated in complex contexts, such as HPE. Qualitative methods, and often related interpretivist worldviews, can help elucidate the modeling of constructs for which scales are developed. Applying these scales in practice can, in turn, inform theory development and further qualitative exploratory studies, creating an iterative process. In other words, quantitative methods frequently build on observations generated through qualitative methods, while qualitative methods provide deeper insight into patterns identified through quantitative research. The value of applying qualitative research methods to psychometrics is arguably most visible in establishing content validity, where expert opinions—including the lived experiences of the population of interest—help identify the most suitable items for a scale. The use of critical inquiry approaches may further strengthen content validity claims by enabling researchers to demonstrate a critical examination and justification of who qualifies as an “expert” on the topic of interest.

**FIGURE 3:**
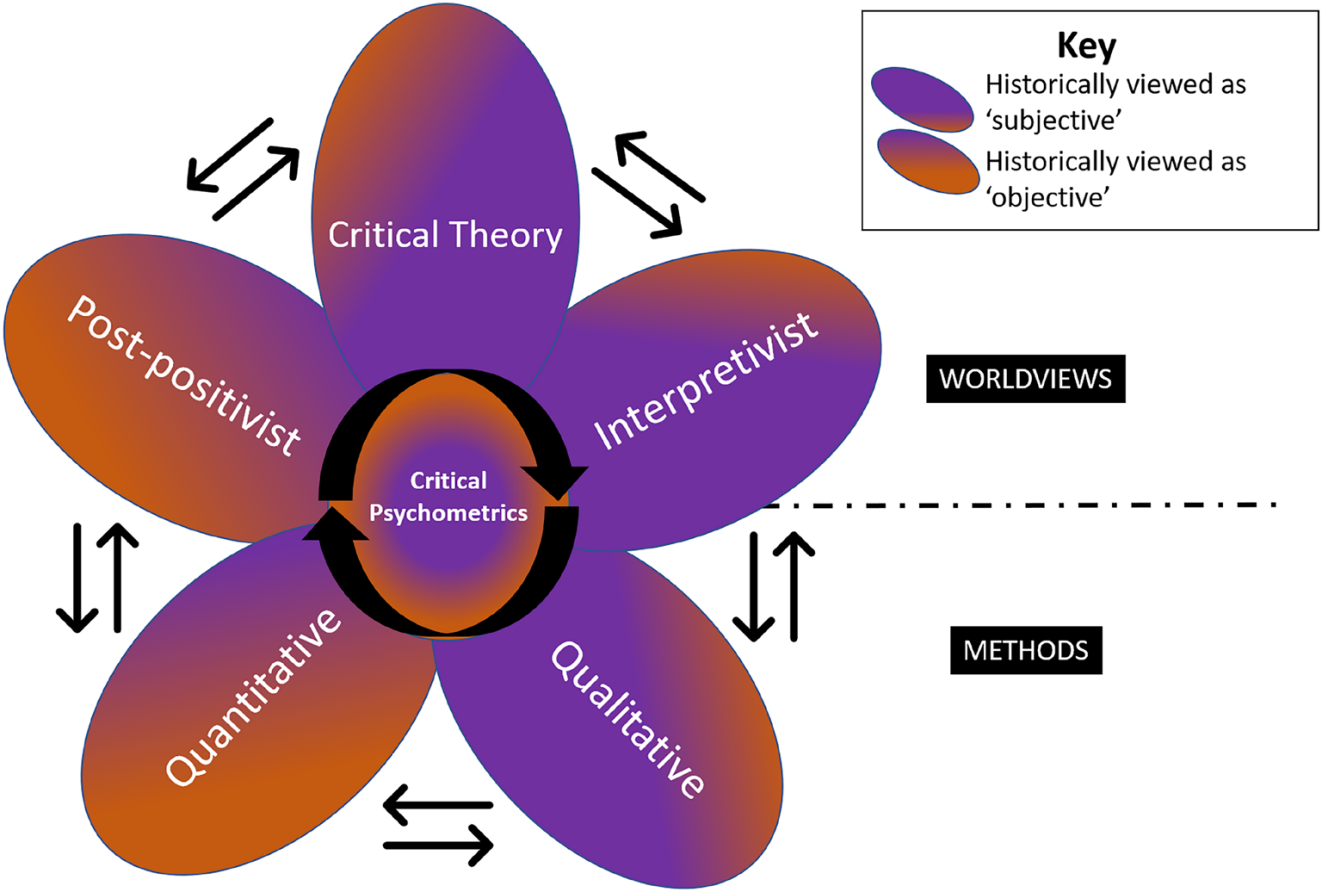
Critical psychometrics—A proposed next step in psychometrics evolution. While historical views of psychometrics portrayed worldviews and methods linearly and as distinct entities, with one being subjective and the other being objective, critical psychometrics raises the visibility that historically subjective worldviews and methods (e.g., interpretivist and qualitative) are required to support psychometrically sound scale development (e.g., post‐positivist and quantitative) and that the outputs of these scales, in turn, influence the interpretivist/qualitative approaches. By integrating critical inquiry into this updated psychometrics approach, we can further enhance the quality and impact of psychometric scales.

We suggest that the next evolution of psychometrics is “critical psychometrics,” where psychometricians engage critical and interpretivist approaches more fully during scale development to consider power dynamics, gender disparities, and socio‐cultural differences as an integral part of validity and reliability. In this psychometric evolution, worldviews and methods historically positioned at opposing ends of a linear continuum would, instead, be considered as interconnected. Such an approach would benefit both scale development and those impacted by such scales (Figure 3). Furthermore, critical psychometrics would request that researchers provide greater transparency in research translation decision‐making, challenging the traditional framing of translating findings from the perspective of a deficit lens (e.g., intolerance or unintelligence) to one of strength. Our goal in writing this article is to challenge researchers to be more transparent and aware of all elements of the scale development and implementation process and to take a more holistic view of psychometrics; a view that sees the interconnectedness of research paradigms, worldviews and methodologies.

### Enhanced transparency about the interdependence of worldviews, methods, and methodologies

Ultimately, an expanded and more nuanced discourse related to scale development, starting with removing assumptions about the relationship between psychometrics and worldviews, is warranted. Indeed, recent work about the role that perspective‐taking can have in health professions research suggests that “it is important to think about how our own perspective affects what we believe and to question our assumptions—embracing the notion that knowledge inherently arises from particular perspectives”.^79^ ^(pp1078–1079)^ There is also research illustrating the value of interrogating and questioning our underlying assumptions and worldviews in programmatic assessment^80^ and assessment in general.^81^

We support these calls for a more robust and vocal movement toward one that emphasizes explicit researcher statements about which worldviews were engaged and at which stage in the research process each was applied—regardless of methods used. Historically, worldviews have been implied in the field of psychometrics as implicitly tied to positivism. A researcher engaging in uncertainty tolerance scale development could—for instance—state that the physician's reaction to the uncertainty tolerance scale,^82^ a scale purported to measure uncertainty tolerance, was developed using CTT from a positivist worldview. This statement can only be assumed today, as the authors did not explicitly state their worldviews or approach. We, as an authorship team, are encouraging researchers to be upfront and transparent about their worldviews and research approaches in all (not just some) psychometric studies. Furthermore, to enhance the recognition that all research paradigms are relevant to psychometrics, researchers should consider also acknowledging the relevant qualitative studies upon which the scales were developed. Such open and honest communications would reduce assumptions about the research process and potentially lead to enhanced interpretations and implications of the research in the manner more in line with the researchers' intent. In support of this, we acknowledge that this work was developed in collaboration with a team of researchers with diverse worldviews, from positivist to interpretivist.

## CONCLUSION

While the prevailing viewpoint is that approaches to psychometrics in health professions education and research rely on positivist worldviews, other relevant worldviews (including post‐positivism, interpretivism, and critical inquiry) have a place in contemporary psychometrics. We encourage the psychometrics field to consider challenging prevailing ideologies in the field and consider alternative worldviews and the value that this diversity brings to the goals of the research process. As the field of psychometrics evolves, we encourage scale developers and those implementing scales to be transparent about their worldview when undertaking psychometric research—and to refrain from placing a value judgment on the quality and rigor of research simply based on the worldview applied and the methods undertaken.

## AUTHOR CONTRIBUTIONS

**Michelle D. Lazarus:** Conceptualization; writing – original draft; writing – review and editing. **Mahbub Sarkar:** Conceptualization; writing – original draft; writing – review and editing. **Claire Palermo:** Conceptualization; writing – original draft; writing – review and editing. **Sze‐Ee Soh:** Writing – original draft; writing – review and editing. **Melanie K. Farlie:** Conceptualization; writing – original draft; writing – review and editing.
